# Outbreak of Hepatitis B Virus Infections Associated with Assisted Monitoring of Blood Glucose in an Assisted Living Facility–Virginia, 2010

**DOI:** 10.1371/journal.pone.0050012

**Published:** 2012-12-26

**Authors:** Thomas John Bender, Matthew E. Wise, Okey Utah, Anne C. Moorman, Umid Sharapov, Jan Drobeniuc, Yury Khudyakov, Marielle Fricchione, Mary Beth White-Comstock, Nicola D. Thompson, Priti R. Patel

**Affiliations:** 1 Virginia Department of Health, Richmond, Virginia, United States of America; 2 Epidemic Intelligence Service Program, Office of Surveillance, Epidemiology, and Laboratory Services, CDC, Atlanta, Georgia, United States of America; 3 Division of Healthcare Quality Promotion, CDC, Atlanta, Georgia, United States of America; 4 Division of Viral Hepatitis, CDC, Atlanta, Georgia, United States of America; 5 Drexel University College of Medicine, Philadelphia, Pennsylvania, United States of America; Duke University, United States of America

## Abstract

**Introduction:**

In January 2010, the Virginia Department of Health received reports of 2 hepatitis B virus (HBV) infections (1 acute, 1 chronic) among residents of a single assisted living facility (ALF). Both infected residents had diabetes and received assisted monitoring of blood glucose (AMBG) at the facility. An investigation was initiated in response.

**Objective:**

To determine the extent and mechanism of HBV transmission among ALF residents.

**Design:**

Retrospective cohort study.

**Setting:**

An ALF that primarily housed residents with neuropsychiatric disorders in 2 adjacent buildings in Virginia.

**Participants:**

Residents of the facility as of March 2010.

**Measurements:**

HBV serologic testing, relevant medical history, and HBV genome sequences. Risk ratios (RR) and 95% confidence intervals (CIs) were used to identify risk factors for HBV infection.

**Results:**

HBV serologic status was determined for 126 (91%) of 139 residents. Among 88 susceptible residents, 14 became acutely infected (attack rate, 16%), and 74 remained uninfected. Acute HBV infection developed among 12 (92%) of 13 residents who received AMBG, compared with 2 (3%) of 75 residents who did not (RR  = 35; 95% CI, 8.7, 137). Identified infection control breaches during AMBG included shared use of fingerstick devices for multiple residents. HBV genome sequencing demonstrated 2 building-specific phylogenetic infection clusters, each having 99.8–100% sequence identity.

**Limitations:**

Transfer of residents out of the facility prior to our investigation might have contributed to an underestimate of cases. Resident interviews provided insufficient information to fully assess behavioral risk factors for HBV infection.

**Conclusions:**

Failure to adhere to safe practices during AMBG resulted in a large HBV outbreak. Protection of a growing and vulnerable ALF population requires improved training of staff and routine facility licensing inspections that scrutinize infection control practices.

## Introduction

In 1990, the Centers for Disease Control and Prevention (CDC) reported the first documented outbreak of 26 cases of acute hepatitis B virus (HBV) infections in the United States that was attributed to reuse of fingerstick devices for capillary blood sampling on multiple patients [1]. Public health authorities identified that nursing staff on an inpatient hospital ward had performed blood glucose monitoring on successive patients without changing disposable lancet end-caps between each patient. Findings of the investigation prompted CDC and the Food and Drug Administration (FDA) to recommend reusable fingerstick devices be dedicated for single patient use [2], [3].

Outbreaks of HBV infections associated with infection control lapses during assisted monitoring of blood glucose have continued [4], [5], [6], [7] and are reported with increasing frequency [8], [9], [10], [11]. In describing these recurring outbreaks, CDC introduced the term “assisted monitoring of blood glucose” (AMBG) [12] as a variant to self-monitoring of blood glucose, the practice routinely self-performed by persons with diabetes. In recent years, such outbreaks have been identified predominantly among older persons residing in assisted living facilities (ALFs). During 1996–2011, there were 17 outbreaks in ALFs, all related to AMBG. These outbreaks resulted in 128 outbreak-associated cases of acute HBV infection among >1250 persons screened [8], [11]. One outbreak included 6 hepatitis-associated deaths among 8 acute HBV cases [10].

HBV is transmitted by percutaneous or mucosal exposure to blood or bodily fluids from an infected person. HBV persists in an infectious state on surfaces and equipment for ≥7 days [13]. Fingerstick devices and blood glucose meters frequently become contaminated with blood [8], [14]. Even if the lancet in a fingerstick device is changed after each use, contamination of the device barrel can result in blood exposure among subsequent patients [12], [15], [16], [17]. During high viral replication activity, an infected person’s blood can contain 10^6^–10^10^ IU/mL HBV DNA [18], [19], [20], [21].

Acute and chronic HBV infections are reportable conditions in Virginia, and state regulations require that these infections be reported to the Virginia Department of Health (VDH) by physicians, directors of laboratories, persons in charge of medical facilities, and persons in charge of ALFs, among others. In January 2010, the VDH received a report of a woman with newly diagnosed acute HBV infection detected through routine HBV screening performed at an outpatient hemodialysis facility. The hemodialysis facility was examined as a potential venue of HBV transmission, but no other infected patients were identified. While investigating this first case, VDH received a report of a man with chronic HBV infection identified during a hospitalization unrelated to HBV. Although the majority of reports of chronic HBV infection do not receive close scrutiny, the VDH’s local epidemiologist noticed a common street address and determined these persons were residents of the same ALF. Both were asymptomatic and aged >50 years. VDH began an investigation with assistance from CDC to determine the number of ALF residents infected with HBV, identify modes of transmission, and implement control and prevention measures.

Ethical review was not deemed necessary for the investigation and control activities documented in this report. This study was exempt from Institutional Review Board approval as it is a description of an outbreak and response that had already occurred.

## Methods

### Setting

The ALF provides long-term residential care to adults who need assistance or supervision with activities of daily living. Most residents had neuropsychiatric disorders. Investigators observed residents’ living environments, and met with the facility administrator and staff to learn about personal and medical services received by residents at the ALF and elsewhere (e.g., adult day care or hemodialysis facility).

### Infection Control Assessment and Record Review

Infection control practices at the facility were assessed by CDC and VDH investigators through review of facility policies on infection control and direct observation of staff practices. Assessments focused on practices where opportunities existed for blood exposure. Using a checklist to document observations, we reviewed staff hand hygiene and glove use, medication preparation and administration practices, and AMBG practices in each of the ALF’s two buildings on at least three separate occasions during both morning and evening medication administration periods. Observation of each medication administration period lasted approximately 20–30 minutes with residents lined up at the entrance of the medication station to received medication and/or AMBG. Staff and resident interviews were performed to attempt to collect information about resident HBV-related risk behaviors (e.g., sexual contact, injection drug use, and sharing of personal care items). Using a questionnaire, separate interviews were conducted with each of the residents with acute or chronic infections. Staff interviews were conducted informally (without use of a questionnaire), and questions were focused on those residents well known to each particular staff member.

We examined facility records for all current residents and those discharged within the previous 6 months to document receipt of medical services that can create an opportunity for blood exposure. We obtained additional risk exposure information by reviewing blood glucose measurements to document AMBG; prescription orders for injected medications (e.g., insulin, which is administered subcutaneously, or medroxyprogesterone, which is administered intramuscularly); resident transportation records for treatment in hemodialysis facilities; and listings of residents who received podiatry services.

### Serologic Testing

Serologic testing was conducted to define the cohort at risk for experiencing acute infection. We collected blood from ALF residents who consented to testing or whose conservators provided consent. Follow-up testing occurred approximately 5 months later. Specimens were tested for hepatitis B surface antigen, total- and IgM-antibody to hepatitis B core antigen, and antibody to hepatitis B surface antigen by using VITROS^®^ ECi Immunodiagnostic System (Ortho-Clinical Diagnostics, Inc., Rochester, NY). Residents were classified as acutely infected, chronically infected, immune, or susceptible to infection [22]. Acute HBV infection was defined as either both positive total anti-HBc and IgM anti-HBc or a seroconversion from hepatitis B surface antigen (HBsAg) negative to positive documented between December 2009 and August 2010. Chronic infection was defined by positive total core antibody (anti-HBc), positive HBsAg, and negative surface antibody (anti-HBs). Immunity to HBV infection was defined as positive for anti-HBs (≥10mg/IU) and either negative (vaccinated) or positive (past HBV infection) for total anti-HBc. Susceptible to infection was defined as negative for all HBV serologic markers.

### Retrospective Cohort Study

A retrospective cohort study was performed to identify risk factors associated with developing acute HBV infection. The study cohort included ALF residents (as of March 1, 2010) who were uninfected and at risk for experiencing acute infection before the investigation (i.e., residents chronically infected or immune were excluded). All statistical analyses were performed by using SAS^®^ 9.2 (SAS Institute, Inc., Cary, NC). We calculated an overall attack rate for acute HBV infection and risk ratios (RRs) and 95% confidence intervals (CIs) for each risk factor assessed.

### HBV DNA Sequence Analysis

The relatedness among virus obtained from resident specimens with detectable HBV DNA was assessed by genotyping and sequencing of the full viral genome. Genotyping was based on the HBV S-gene sequence (nucleotides 222–656) [23]. Whole-genome sequencing was conducted as published elsewhere [24]. Viral sequences from infected residents were compared with each other and with selected viral sequences from the CDC Division of Viral Hepatitis library, which were used as a reference group. Phylogenetic analysis with MEGA5 (Biodesign Institute, Tempe, AZ) used the neighbor-joining method to construct a dendrogram [25], [26].

## Results

### Setting

The ALF had 160 beds in 2 adjacent buildings with 2–3 residents/room. Among 139 residents present in March 2010, median age was 59 years (range: 28–93 years); 57 (41%) were female; 82 (59%) were black; and median duration of residence at the facility was 1,645 days (range: 1–8,364 days). Neuropsychiatric diagnoses were documented for 129 (93%) residents and included schizophrenia (95), other psychiatric diagnoses (45), mental retardation (21), seizure disorder (20), substance abuse (13), dementia (11), stroke (7), and traumatic brain injury (5). A majority of residents were dependent on Medicaid and Supplemental Security Income to pay expenses.

Among 36 ALF employees, 3 were trained to perform AMBG and administer insulin injections, including a licensed practical nurse, a certified nursing assistant, and a registered medication aide. However, AMBG was also performed routinely by direct care staff, who are unlicensed and comprise the majority of ALF employees. Staff usually rotated between buildings, but some worked routinely in a single building. Phlebotomy, wound care, and podiatry services were performed by contracted providers who visited the facility periodically and not by ALF staff. Antipsychotic medication injections were administered by psychiatrists.

### Infection Control Assessment and Record Review

The ALF had no written infection control policy. We observed lapses in infection control practices. When observed performing AMBG or injecting insulin, staff sometimes failed to wash hands or change gloves between residents. Staff struggled to don gloves, suggesting glove use was not routine practice, especially for staff with long fingernails.

Staff in each building performed AMBG for residents, and blood samples were routinely obtained by using a single ACCU-CHEK^®^ Softclix (F. Hoffman-La Roche, Ltd., Basel, Switzerland) reusable lancet-holding fingerstick device. A new lancet was inserted for each fingerstick, but the fingerstick device, which is intended for personal use, was used for multiple residents. Blood glucose readings were obtained with a single meter device in each building. The meter was not cleaned or disinfected between uses. No resident performed self-monitoring of their own blood glucose.

Staff and resident interviews and record review produced limited information about resident HBV-related risk behaviors. Sexual contact was uncommon in the facility and unknown to have occurred among residents uninfected before the investigation. Injection-drug use and sharing of personal care items could not be reliably assessed.

### Serologic Testing

**Figure 1:**
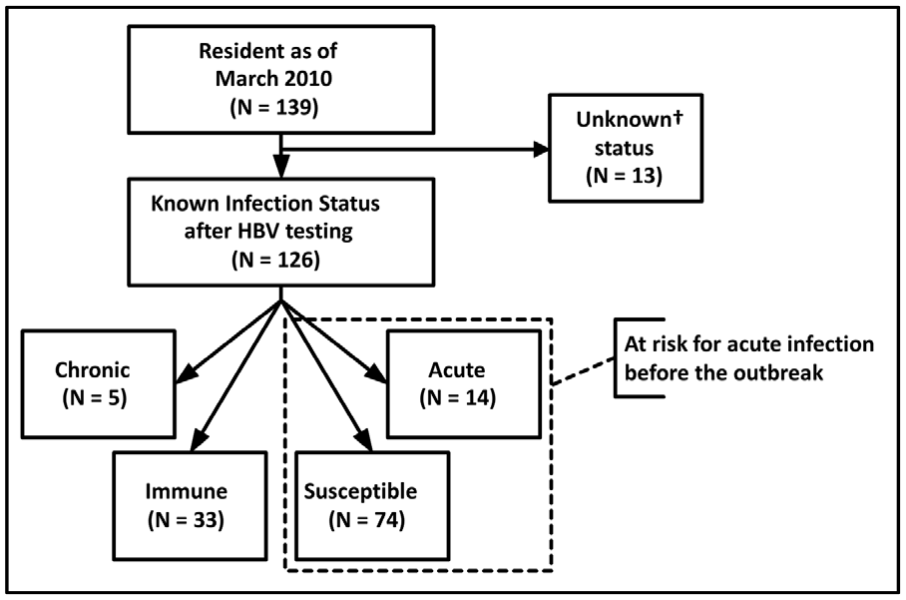
Flowchart Depicting Results of Serological Testing for Hepatitis B Virus Among Assisted Living Facility* Residents and Identifying Members of Cohort for Risk Factor Analysis. * Assisted living facility (ALF).

HBV serologic status was determined for 126 residents (91%) (Figure 1). Of these, 5 (4%) had chronic infection (2 were known to be infected before the investigation). Thirty-three (26%) were immune (24 had evidence of past infection; 9 vaccinated). Fourteen residents (11%) had acute infection and 74 (59%) remained susceptible to infection. Two (14%) residents with acute infection were hospitalized with hepatitis symptoms, and 12 (86%) were asymptomatic and diagnosed on the basis of serologic screening. Serologic status was unknown for 10 ALF residents who refused serologic testing and for 3 ALF residents whose results were ambiguous.

### Retrospective Cohort Study

Among 88 residents included in the retrospective cohort study, the attack rate was 16% (Figure 1). Residents who experienced acute infection were similar to the total cohort in terms of age, sex, and race (Table 1). Mean length of stay at the ALF was shorter for acutely infected residents than for the total cohort. Among 14 acutely infected residents, 11 (79%) lived in building 2; 12 (86%) had diabetes; 12 (86%) had received AMBG; 7 (50%) had received injected medications; 9 (64%) had received podiatry services; and 1 (7%) had received hemodialysis.

The risk for experiencing acute HBV infection was higher among residents who received AMBG ([RR], 35; 95% [CI], 8.7–137) or injected medications ([RR], 3.0; 95% [CI], 1.2–7.6) compared with residents who were not exposed to these procedures (Table 2). Stratifying injected medications by receipt of AMBG revealed that all 7 acutely infected residents who received injected medications also had received AMBG (Table 2). Acute HBV infection developed among 12 (92%) of 13 residents who received AMBG, compared with 2 (3%) of 75 residents who did not (RR  =  35; 95% CI, 8.7, 137).

**Table 1:** Characteristics of the Cohort of 88 Assisted Living Facility Residents, Virginia – March 2010.

	Acute	Total
	(n = 14)	(n = 88)
Demographic characteristic		
Median age in years (Range)	64 (46–84)	59 (31–93)
Women, no. (%)	9 (64)	40 (45)
Black, no. (%)	9 (64)	45 (51)
Mean length of stay (days)	1,126	1,615
(range)	(70–4,170)	(1–8,364)
Building 1, n (%)	3 (21)	44 (50)
Building 2, n (%)	11 (79)	44 (50)
Diabetes, n (%)	12 (86)	13 (15)
Medical service received, n (%)		
AMBG*	12 (86)	13 (15)
Injected medications	7 (50)	22 (25)
Podiatry	9 (64)	39 (44)
Hemodialysis	1 (7)	1(1)

* Assisted monitoring of blood glucose.

**Table 2:** Assessment of Assisted Living Facility Resident Risk Factors for Hepatitis B Virus Infection, Virginia – March 2010.

Risk Factor	Acute Infections/Total Number		Risk Ratio
	at Risk for Acute Infection		(95% CI)†
	at Beginning of Outbreak* (%)		
	Exposed	Unexposed	
AMBG‡	12/13 (92)	2/75 (3)	35 (8.7, 137)
Injected medications	7/22 (32)	7/66 (11)	3.0 (1.2, 7.6)
AMBG‡ (+)	7/7 (100)	5/6 (83)	1.2 (0.8, 1.7)
AMBG‡ (−)	0/15 (0)	2/60 (3)	1.0 (0.98, 1.1)
Podiatry	9/39 (23)	5/49 (10)	2.3 (0.8, 6.2)
AMBG‡ (+)	8/9 (89)	4/4 (100)	0.9 (0.7, 1.1)
AMBG‡ (−)	1/30 (3)	1/45 (2)	1.5 (0.1, 23)

* Uninfected and at risk for experiencing acute infection prior to the outbreak (i.e., subsequently determined by serologic testing to have become acutely infected or remained susceptible to infection).

† Confidence interval.

‡ Assisted monitoring of blood glucose.

### HBV DNA Sequence Analysis

**Figure 2:**
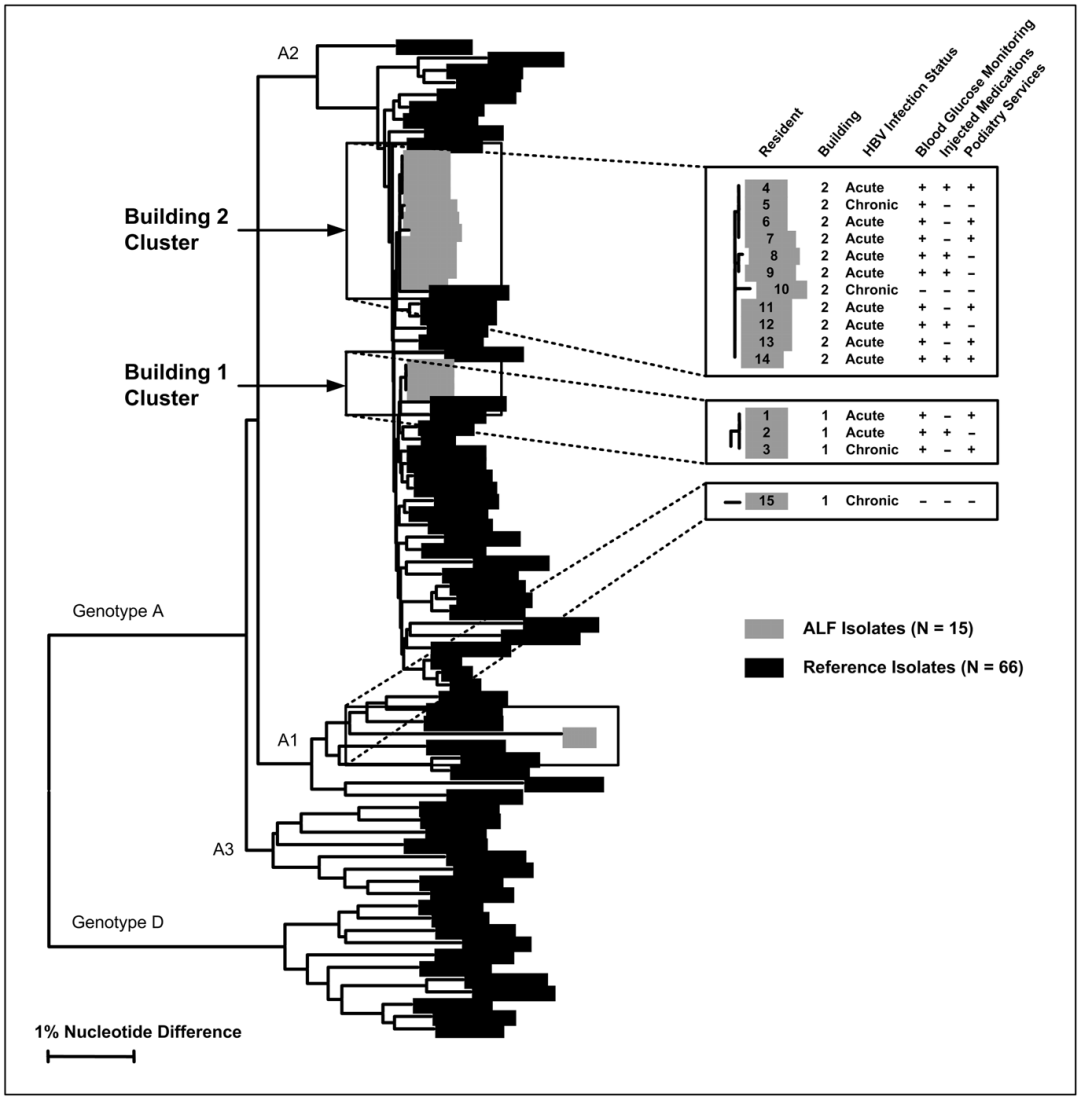
Dendrogram Illustrating the Genetic Relatedness of the Hepatitis B Virus* DNA Sequences from Assisted Living Facility† Residents with Acute or Chronic Infections. * Hepatitis B virus (HBV). † Assisted living facility (ALF).

HBV DNA was available for genotyping and sequencing for 11 (79%) of 14 residents with acute infection and 4 (80%) of 5 residents with chronic infection. Among these 15 residents, 14 had HBV belonging to subgenotype A2; 1 resident with chronic HBV infection who did not receive blood glucose monitoring had HBV that belonged to A1 subgenotype. Sequences from residents infected with HBV subgenotype A2 formed 2 genetic clusters, each with 99.8–100% sequence identity (Figure 2). Each cluster was comprised only of specimens from infected persons residing in the same building.

The building 1 cluster included specimens from 1 resident with chronic infection and 2 residents with acute infection; all 3 received AMBG. The chronically infected resident (whose HBV DNA concentration was 2×10^9^ IU/mL) had resided at the facility for 9 years. The building 2 cluster included specimens from 2 residents with chronic infection and 9 residents with acute infection. Among these 11, a total of 10 residents had received AMBG, including a chronically infected resident (whose HBV DNA concentration was 1.2×10^8^ IU/mL) who had resided at the facility for 1 year.

### Outbreak Control Measures

VDH provided infection control recommendations both to the ALF and to the Department of Social Services, the agency responsible for licensing and inspecting ALFs in Virginia. VDH worked with ALF staff and licensing inspectors to ensure adoption of single-use, auto-disabling lancets and separate glucose meters for each resident needing AMBG. VDH offered susceptible residents the hepatitis B vaccine: 61 of 74 (82%) residents agreed to be vaccinated and completed the 3-dose vaccination series. Only one resident who had diabetes was susceptible to HBV infection, and this resident was discharged from the ALF before receiving the third dose of the 3-dose vaccination series. VDH coordinated with Virginia Commonwealth University to ensure all HBV-infected residents received clinical follow-up and evaluation of therapy for chronic infection.

## Discussion

Investigation of a single acute HBV infection uncovered an outbreak of 14 acute infections among residents of an ALF. The majority of acutely infected residents were asymptomatic, and so this outbreak easily might have gone undetected. Resident-to-resident transmission of HBV most likely occurred during AMBG through use of the same reusable fingerstick devices on multiple residents. Inadequate cleaning and disinfection of glucose meters between residents could have also played a role in transmission. Virtually all (12 of 13) at-risk ALF residents who were receiving AMBG experienced acute HBV infection. The HBV DNA sequences from infected residents were clustered by the building in which they lived; residents with chronic infection also receiving AMBG were identified as the likely source of HBV in each building. Lack of education and the absence of a written infection control policy likely contributed to a permissive culture among staff and an overall lackadaisical approach to preventing infection.

Hepatitis B is a vaccine-preventable disease, and adoption of current vaccination recommendations might have prevented this outbreak. Aware of this and other similar outbreaks, the Advisory Committee on Immunization Practices recommended in 2011 that adults aged 19–59 years with diabetes mellitus (type 1 and type 2) be vaccinated against HBV as soon as possible after a diagnosis of diabetes is made [27]. Adults aged ≥60 years with diabetes may be vaccinated at the discretion of the treating clinician after assessing their risk and the likelihood of an adequate immune response to vaccination. However, vaccination alone is unlikely to eliminate bloodborne pathogen (e.g., HBV) transmission risk in ALFs and should not be a subsitute for adequate infection control.

HBV transmission occurred at this ALF because CDC’s long-standing recommendations for preventing bloodborne pathogen transmission during AMBG were not followed [2], [5], [7], [28]. Despite efforts to improve AMBG practices in Virginia and elsewhere [15], outbreaks associated with AMBG have continued to occur [4], [8], [9], [10]. Inadequate infection control practices during AMBG have been identified and associated with disease transmission in other settings, including nursing homes, ambulatory surgery centers, health centers, and health fairs [15], [17], [29], [30], [31], [32]. Persons responsible for providing AMBG, ALFs or in any setting, should be trained in and adhere to CDC’s infection control recommendations. Because of the widespread under-appreciation of the risks for bloodborne pathogen transmission when providing AMBG, CDC issued updated guidance on infection control practices during AMBG and insulin administration in 2011 [28]. CDC recommends single-use retractable safety lancets rather than reusable fingerstick devices when performing AMGB [28].

Outbreaks of this type are now far less likely to occur in nursing homes, which are subject to federal regulation by the Centers for Medicare and Medicaid Services (CMS). In 2008, recommendations for safely performing AMBG were incorporated into CMS conditions for coverage that dictate the standard of care for nursing homes [33]. Use of reusable fingerstick devices for multiple persons is a practice that can result in a nursing home’s loss of billing privileges. Unlike nursing homes, however, ALFs are licensed by states, and state regulations are variable [34]. The frequency of HBV outbreaks at ALFs might reflect this inconsistent oversight and that provision of medical services has not been a primary focus of these facilities [35]. ALFs have limited or no physician oversight, hire few licensed nurses, are primarily staffed by paraprofessionals (e.g., direct care staff and medication aides), and experience substantial staff turnover [36], [37]. Substantial turnover compromises the ability to train staff and limits impact of educational interventions.

One limitation of this study was the outbreak might have been larger than we describe. Residents with evidence of past HBV infection might have acquired the infection in the ALF. Residents transferred from the ALF before this investigation also might have become infected while living there. A previous resident was identified as the source of HBV transmission at another ALF, where infections occurred among residents receiving AMBG and HBV DNA sequences matched the building 2 cluster [4]. Second, our efforts to assess other resident risk factors for HBV infection beyond those included in this analysis were limited because resident interviews provided insufficient information. Resident interview responses were sometimes unreliable because of residents’ neuropsychiatric disorders; some refused to be interviewed; and some were deceased. Nevertheless, our epidemiologic investigation clearly identified the receipt of AMBG as the major risk factor for infection. Although receipt of injected medications was also a risk factor, this association was confounded by AMBG. The 2 acute infections identified in residents who did not receive AMBG might be attributed to secondary transmission through sexual or household contact with an infected resident [38].

## Conclusions

Physicians diagnosing patients with acute HBV should report these cases to public health authorities and carefully consider the role of health care exposures, especially among older adults or others who do not report traditional risk factors for infection such as high-risk sexual behaviors and injection-drug use [39]. An acute HBV infection occurring in a long-term care resident represents a warning signal for possible medical transmission that deserves thorough investigation [40].

ALF resident populations will likely continue to grow. The U.S. population aged ≥65 years is projected to double, exceeding 70 million by 2030 [41]. A substantial proportion of this population has diabetes, many of whom will undergo blood glucose monitoring [42]. Therefore, broad adoption of policies to eliminate preventable spread of HBV during AMBG will be essential. The safe provision of AMBG requires adherence to recommended infection control standards irrespective of the care setting. Prevention efforts will be aided by improvements in training and education of staff responsible for providing AMBG services, oversight of staff practices, and licensing inspections of ALFs that include evaluation of AMBG. Following a similar outbreak [10], legislation was passed in North Carolina to increase educational requirements for unlicensed staff in ALFs, increase infection control requirements for ALFs, and require public health authorities to assess infection control in ALFs annually [43]. North Carolina’s approach can serve as a model for other states.
